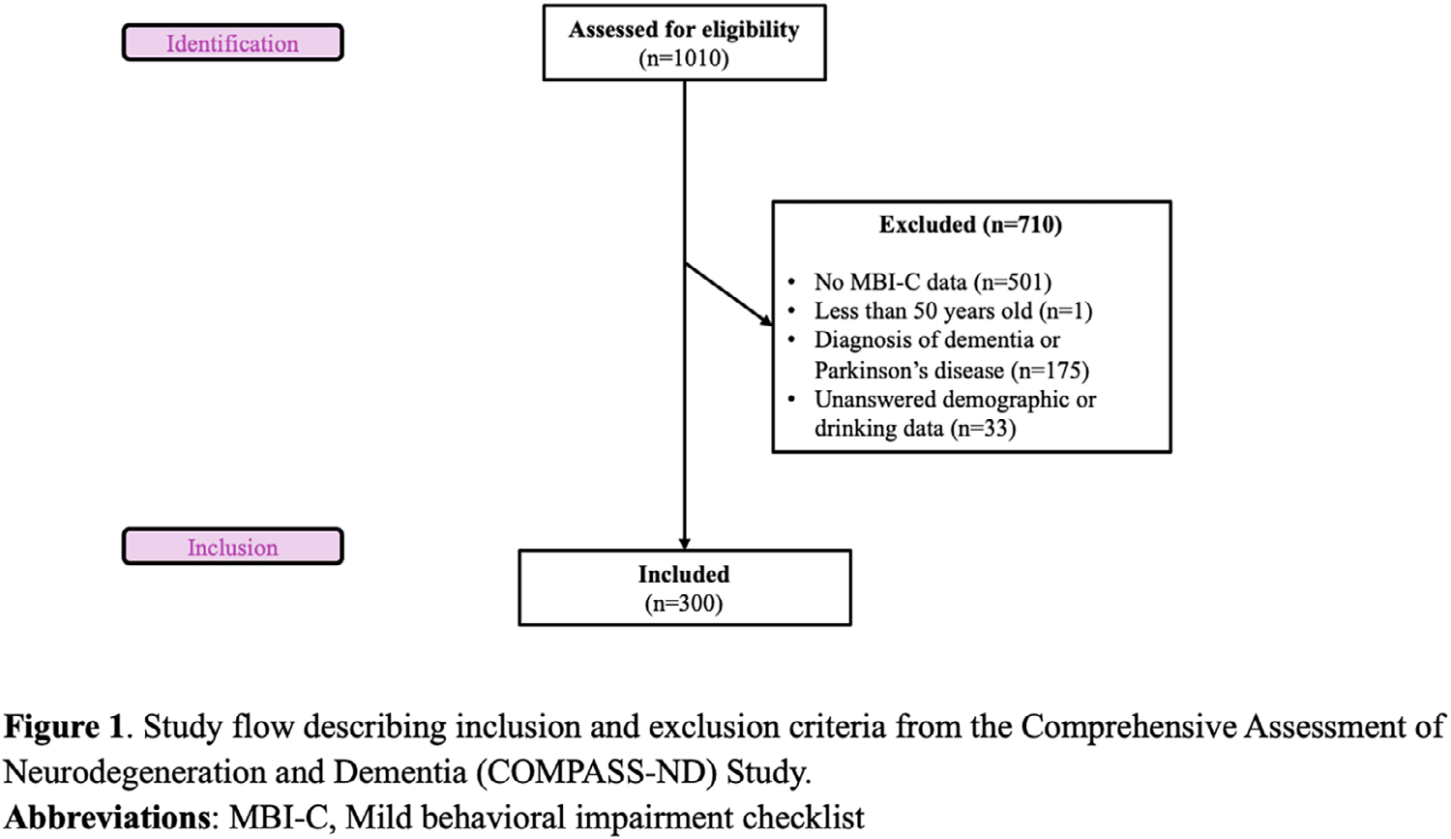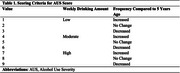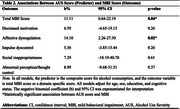# Alcohol Consumption and Mild Behavioral Impairment

**DOI:** 10.1002/alz70860_099830

**Published:** 2025-12-23

**Authors:** Ibadat Warring, Dylan X. Guan, Maryam Ghahremani, Eric E. Smith, Zahinoor Ismail

**Affiliations:** ^1^ University of Calgary, Calgary, AB, Canada; ^2^ Hotchkiss Brain Institute, University of Calgary, Calgary, AB, Canada

## Abstract

**Background:**

Mild behavioral impairment (MBI) is a neuropsychiatric syndrome that identifies individuals at high risk for dementia. Alcohol consumption is a modifiable risk factor for dementia, yet its relationship with MBI remains unexplored. This study examines the association between alcohol consumption and MBI symptom severity in dementia‐free older adults, hypothesizing that greater alcohol consumption would correlate with more severe MBI symptoms.

**Method:**

Cross‐sectional data for 300 participants aged ≥50 years were obtained from the Comprehensive Assessment of Neurodegeneration and Dementia (COMPASS‐ND) study (Figure 1). MBI was assessed using the Mild Behavioral Impairment Checklist (MBI‐C), with total and domain‐specific scores (decreased motivation, affective dysregulation, impulse dyscontrol, social inappropriateness, and abnormal perception/thought content). Alcohol consumption was operationalized using the Alcohol Use Severity (AUS) score, a composite measure of weekly drinking amount and frequency relative to five years ago (Table 1). Associations between AUS and MBI‐C scores were analyzed using zero‐inflated negative binomial models, adjusted for age, sex, education, and cognitive status. A sex*AUS interaction term was included in the models, and if non‐significant, sex was only included as a covariate.

**Result:**

Participants (mean age: 70.9 ± 6.4 years, 55.3% female) included 87 classified as MBI+ and 213 as MBI‐. Every one‐unit increase in AUS score was associated with an estimated 11.11% higher MBI‐C score (95% CI: 0.64–22.19, *p* = 0.04) (Table 2). The sex*AUS score interaction term was not significant (*p* = 0.33). The affective dysregulation domain was also significantly associated with a 14.10% higher MBI‐C score for each one‐unit increase in the AUS score (95% CI: 2.26–27.30, *p* = 0.02); the domains of decreased motivation (*p* = 0.26), impulse dyscontrol (*p* = 0.26), social inappropriateness (*p* = 0.61), and abnormal perception/thought content (*p* = 0.37) were not.

**Conclusion:**

More severe alcohol consumption is associated with greater MBI symptom severity, particularly in the affective dysregulation domain. These findings highlight the potential impact of alcohol consumption on neuropsychiatric symptoms, emphasizing the importance of addressing alcohol use in dementia prevention strategies. Future studies should investigate underlying biological mechanisms and explore longitudinal associations between alcohol consumption, MBI, and dementia risk.